# Candidate Multi-Epitope Vaccine against Corona B.1.617 Lineage: In Silico Approach

**DOI:** 10.3390/life12111715

**Published:** 2022-10-27

**Authors:** Mohamed G. Seadawy, Abdel Rahman N. Zekri, Aya A. Saeed, Emmanuel James San, Amr M. Ageez

**Affiliations:** 1 Biological Prevention Department, Chemical Warfare, 4.5 km Suez-Cairo Rd, Almaza, Cairo 11351, Egypt; 2 National Cancer Institute, Cairo University, Giza 12613, Egypt; 3 KwaZulu-Natal Research Innovation and Sequencing Platform, School of Laboratory Medicine & Medical Sciences, University of KwaZulu-Natal, Durban 4001, South Africa; 4 Faculty of Biotechnology, MSA University, 6 October City 12451, Egypt

**Keywords:** COVID-19, D614G mutation, P681R mutations, epitope prediction, in silico vaccine design

## Abstract

Various mutations have accumulated since the first genome sequence of SARS-CoV2 in 2020. Mutants of the virus carrying the D614G and P681R mutations in the spike protein are increasingly becoming dominant all over the world. The two mutations increase the viral infectivity and severity of the disease. This report describes an in silico design of SARS-CoV-2 multi-epitope carrying the spike D614G and P681R mutations. The designed vaccine harbors the D614G mutation that increases viral infectivity, fitness, and the P681R mutation that enhances the cleavage of S to S1 and S2 subunits. The designed multi-epitope vaccine showed an antigenic property with a value of 0.67 and the immunogenicity of the predicted vaccine was calculated and yielded 3.4. The vaccine construct is predicted to be non-allergenic, thermostable and has hydrophilic nature. The combination of the selected CTL and HTL epitopes in the vaccine resulted in 96.85% population coverage globally. Stable interactions of the vaccine with Toll-Like Receptor 4 were tested by docking studies. The multi-epitope vaccine can be a good candidate against highly infecting SARS-CoV-2 variants.

## 1. Introduction

The emergence of the Severe Acute Respiratory Syndrome Coronavirus 2 (SARS-CoV-2) which causes COVID-19 has led to a high mortality rate all over the world. The virus attacks the vital organs of the body and leads to fatal respiratory distress. SARS-CoV-2 belongs to a group of viruses known as human coronaviruses [1]. Human coronavirus is a member of the *Coronaviridae* family that infects the respiratory tract of humans. They can be classified into seven genera of coronaviruses. Four human corona viruses namely HCoV-HKU1, HCoV-OC43, HCoV-NL63, and HCoV-229E, can lead to mild or weak respiratory symptoms [2]. The remaining three, Severe Acute Respiratory Syndrome Coronavirus (SARS-CoV), Middle East Respiratory Syndrome Coronavirus (MERS-CoV), and Severe Acute Respiratory Syndrome Coronavirus 2 (SARS-CoV-2), are highly pathogenic with symptoms including high fever, dry cough, fatigue and can lead to severe pneumonia [3].

The virus targets the angiotensin-converting enzyme 2 (ACE2) receptor of the host cell. Spike glycoprotein (S-protein) of CoV binds to the ACE2 receptor and releases its RNA into the cytoplasm of the host cell that is translated into polyproteins and structural protein. The S protein has two subunits; S1 which harbors the receptor-binding domain (RBD) and responsible for the binding to the host ACE2. The second subunit S2 contains the membrane fusion machinery. [4]. Spike protein is the prime choice for vaccine designing because it is involved in receptor recognition, as well as virus attachment and entry. Most approved candidates of COVID-19 vaccines are based upon the Spike (S) protein, which is the target of neutralizing antibodies [5].

Since the first genome sequence of SARS-CoV2 in 2020 many variants with various mutations have emerged and spread worldwide. Five variants were labeled variants of concern (VOCs); Alpha (B.1.1.7), Beta (B.1.351), Gamma (B.1.1.28.1), Delta (B.1.617.2) and Omicron (B.1.1.529). Multiple SARS-CoV-2 variants with substantial features such as increased transmissibility and/or potential immune escape have emerged. During the COVID-19 pandemic, SARS-CoV-2 acquired various mutations. The spike (S) protein D614G mutation, emerged in spring 2020 and become predominant. The D614G-bearing variant has increased viral infectivity and inter-individual transmissibility [6,7].

The corona B.1.617 lineage has emerged in India during 2021. Patients of B.1.617 lineage variants displayed lower efficacy against infection after vaccination. The variant showed more pathogenicity than the prototypic SARS-CoV-2 in the Syrian hamster model. The S protein P681R mutation is the signet mutation of this lineage [8]. The P681R mutation is part of a proteolytic cleavage site for furin and furin-like proteases at the junction of the spike protein receptor-binding (S1) and fusion (S2) domains and is responsible for the higher pathogenicity of the B.1.617.2/Delta variant in vivo [9,10]. There are limited data about the efficacy of the current vaccines against this variant, especially in high-risk populations. Sera from individuals who had received one dose of the Pfizer or the AstraZeneca vaccine showed a very low inhibitory effect against the Delta variant. After receiving two doses of the vaccine, the neutralizing response in 95% of individuals was three to fivefold lower against the Delta variant than against the Alpha variant [11].

Vaccination remains an urgent need to control and eliminate SARS-CoV-2. The use of immunoinformatic tools in the design of vaccines against diseases is cost-effective and convenient. In silico approaches have paved the way to design immunogenic and highly conserved epitopes from viral antigens [12,13,14]. Epitope-based vaccines have many advantages over those produced by conventional methods. They are easier, faster to produce, and do not require microbial culturing. Multi-epitope vaccines do not contain the whole virus, so they are safer, more specific, and more stable. Finally, the design of promiscuous T cell epitopes can bind to multiple alleles inside human population that will ensure the expected immune response among a heterogeneous human population [14]. Many studies have generated epitope-based peptide vaccines of SARS-CoV-2. The multi-epitope vaccine can provoke cellular and humoral responses in human hosts [15,16,17,18].

The current report describes the design of a multi-epitope vaccine against SARS-CoV-2 carrying the D614G and P681R mutations. The spike multi-epitope protein was in silico tested for the induction of cytotoxic T lymphocytes (CTLs), helper T lymphocytes (HTLs).

## 2. Materials and Methods

### 2.1. Retrieval of SEQUENCE of Delta Variant

The amino acid sequence of spike protein of SARS-CoV2 Delta variant was used in this study. The amino acid changes T19R, (V70F*), T95I, G142D, E156-, F157-, R158G, (A222V*), (W258L*), (K417N*), L452R, T478K, D614G, P681R, D950N were introduced into the spike (S) protein of the Wuhan-Hu-1 reference strain (NC_045512.2) [19,20]. The * indicates that mutation of this amino acid can or cannot occur and will not disturb the identification of the Delta variant

### 2.2. Prediction of T Cell Epitope

The NetCTL 1.2 server (http://www.cbs.dtu.dk/services/NetCTL/) (accessed on 15 September 2021)was used to predict CTL epitopes for the spike protein of SARS-CoV-2 Delta variant. The threshold value was set to 0.75 with high sensitivity and specificity. The epitopes were selected based on antigenicity using VaxiJen v2.0 (http://www.ddg-pharmfac.net/vaxijen/VaxiJen/VaxiJen.html) (accessed on 20 September 2021), and immunogenicity using IEDB server (IEDB; http://tools.iedb.org/mhcii/) (accessed on 29 September 2021). [21].

HTL epitopes were predicted using NetMHCII pan 3.2 server (www.cbs.dtu.dk/services/NetMHCIIpan/) [22]. All the selected epitopes were checked using BLASTp (https://blast.ncbi.nlm.nih.gov/Blast.cgi) (accessed on 30 September 2021), against the human genome to discard autoimmunity potentials.

### 2.3. Structural Modeling of Multi-Epitope Vaccine, Refinement, and Validation

The selected CTL and HTL from the spike glycoprotein were joined by the flexible GPGPG linkers to ensure the effective separation of the epitope. The Cholera Toxin B (CTB) adjuvant was added to the N-terminal of the vaccine construct. To induce regulatory immune responses, the 3-D model was generated using the scratch 3Dpro tool [23]. The 3D model is refined by GalaxyRefine [24] and validated by the ProSAweb server [25].

### 2.4. Prediction of Antigenicity, Allergenicity, Immunogenicity and Physiochemical Features

The antigenicity of the multi-epitope vaccine construct was determined using VaxiJen v2.0 with a threshold value of 0.4. AllerTop server (https://www.ddg-pharmfac.net/AllerTOP/) (accessed on 7 December 2021), server and AllergenFP server (https://ddg-pharmfac.net/AllergenFP/) (accessed on 21 December 2021), checked the Allergenicity of the designed vaccine [26]. The ExPASy ProtParam server was used to identify the molecular weight, instability index, isoelectric point, aliphatic index, half-life, and GRAVY score of the vaccine [27].

### 2.5. Population Coverage

The IEDB population coverage analysis tool [28] with default parameters, was used to confirm that the designed vaccine is covering the entire world population. The analysis was performed against HLA alleles (Class I and Class II).

### 2.6. Molecular Docking

Protein-protein docking, rigid docking, was employed using the ClusPro 2.0 server [29]. The TLR4 (PDB ID: 4G8A) was used as the immune receptor. Unstructured terminal residues were removed from the TLR-4 structure through ClusPro 2.0 server. Docked complex showing the lowest energy score was selected for molecular dynamics simulation. The iMOD server (iMODS) was used for investigating the molecular dynamics simulation [30].

### 2.7. Codon Adaptation, In Silico Cloning and Testing the Stability of the Secondary Structure of the Produced mRNA

Codon optimization of the multi-epitope construct was performed by vector builder. The construct was uploaded to the codon adaptation service of vector builder (https://en.vectorbuilder.com/tool/codon-optimization.html) (accessed on 17 October 2022). In silico cloning of the adapted nucleotide sequence into the pET28a (+) expression vector was performed using SnapGene v4.2 software (GSL Biotech LLC, Chicago, IL, USA). The folding of the expressed RNA sequence was performed using RNAfold server [31].

## 3. Results

### 3.1. CTL/HTL Epitope Prediction and Assessment

Both CTL and HTL epitopes have an essential role in the generation of long-lasting adaptive immunity. They have an important role in the induction of humoral and cellular immune responses. HTL epitopes provoke a CD4+ helper reaction, that leads to the induction of CD8+ T-cell memory and the stimulation of B-cells for antibody production [32,33]. The of CTL epitopes (9 mer) was selected using the NetCTL server2.1. The S protein of SARS-CoV-2 Delta variant was scanned for the CTL epitopes. Various immune filters were used to confirm that the predicted epitopes have a strong affinity to MHC class I and class II alleles. The epitopes were tested and been confirmed to be promiscuous, antigenic, and immunogenic. Seven epitopes meeting the previous criteria were selected for construct design (Table 1A). Similarly, the S protein of SARS-CoV-2 Delta variant was scanned for the HTL epitopes. Four epitopes were selected and confirmed to be antigenic, non-allergenic, and non-toxic and selected for construct design (Table 1B). All selected epitopes were checked for autoimmunity against human genome using BLASTp and showed no significance.

### 3.2. Multi-Epitope Vaccine Construct Design

**Figure 1:**
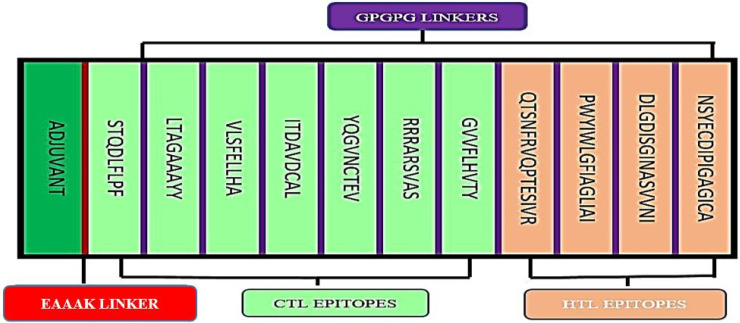
Design of the multi-epitope vaccine construct. The CTL and HTL are in green and light pink, respectively. The epitope was linked by GPGPG linkers (purple). Adjuvant (CBT sequence) was linked by EAAAK linker (red).

The final epitopes were joined together via flexible GPGPG linkers to ensure that the epitope domains are separated and for decreasing the possibility of creating junctional epitopes (Figure 1). The sequence of the multi-epitope vaccine was proceeded by Cholera Toxin β subunit (CTB) sequence to improve the overall immunogenicity of the multi-epitope. CTB is a non-toxic constituent of cholera toxin, showed high affinity to good mono sialo-tetrahexosylganglioside on the surface of the gut epithelium. Moreover, it also has a high affinity to macrophages, B-cells, and dendritic cells. The CTB sequence should lead to high efficiency in activating the human immune system. The CTB was joined to first epitope in the designed construct by EAAAK linker sequence. The molecular weight of the designed vaccine is 29.2 kDa.

### 3.3. Prediction of Immunogenicity, Antigenicity, Allergenicity, and Physicochemical Parameters

VaxiJen v2.0 and ANTIGENpro were used to predict the antigenic property of the multi-epitope vaccine and it showed 0.67 and 0.899, respectively. The immunogenicity of the predicted vaccine was calculated using IEDB server and yielded 3.4. The AllerTop software confirmed that the multi-epitope vaccine is non-allergenic in nature. The physicochemical characteristics of the construct was investigated by the ProtParam. The vaccine construct has a molecular weight of 29.2 kDa. The theoretical isoelectric point (pI) was 6.06. The multi-epitope vaccine has of 282 amino acids, 22 negative charged aa and 19 positive charged aa. The chemical formula of the vaccine is C_1303_H_2025_N_355_O_388_S_10_. It has aliphatic index of 80.32 characteristic of a vaccine candidate that is highly thermostable. The construct should yield a stable protein after expression, as it has instability index of 32.35. The vaccine has a GRAVY index value of -0.063 which reflects the hydrophilic nature of the vaccine. The expected half-life of the vaccine is 30 h in mammalian reticulocyte (in vitro).

### 3.4. Population Coverage Study

The epitopes used in vaccine design were tested for coverage of the covid-19 maximum allele population. Notably, our results showed that the CTL and HTL epitopes used in our design resulted in 96.85% population coverage globally. Population coverage analysis showed that the vaccine should yield a 99.8% coverage for England, 82% for east Africa and 99.3% for Europe. For India and Brazil, that were highly affected by SARS-CoV-2, the lower coverages of 88.89% and 85.39%, respectively were not surprising (Table 2).

### 3.5. D Structure Modeling and Validation of the Multi-Epitope

**Figure 2:**
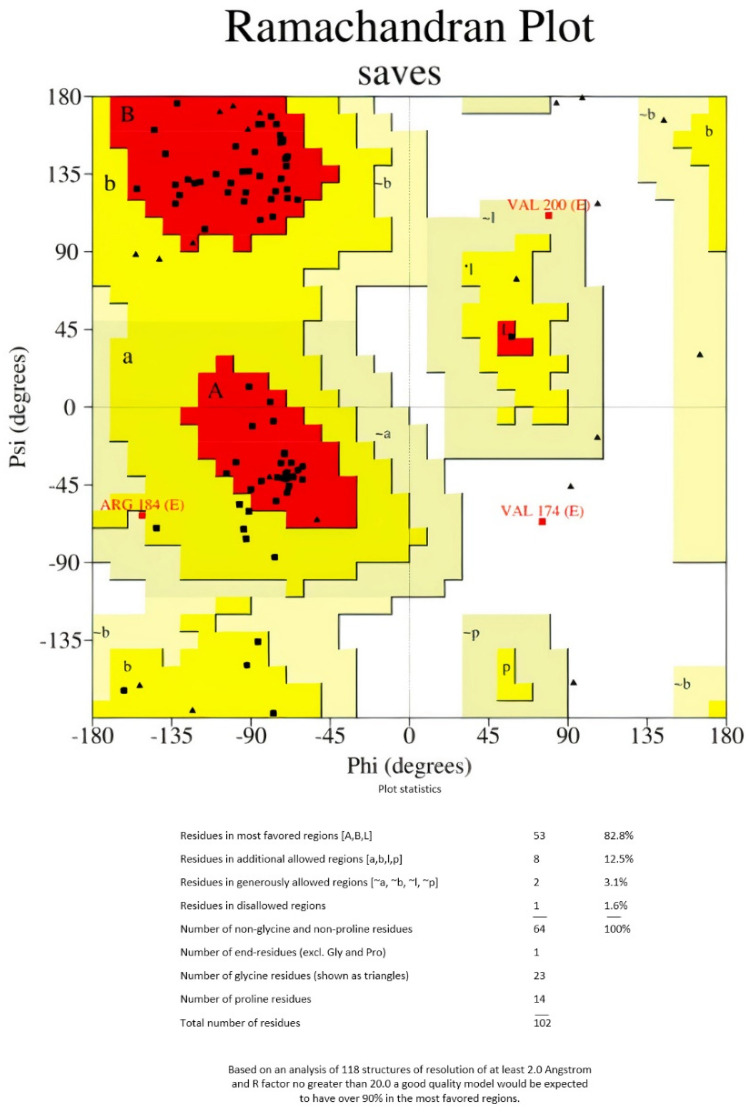
Ramachandran plot of the refined vaccine construct.

As there are no good PDB templates to guide the protein structure prediction process, the 3Dpro tool was used to model the 3D structure of the multi-epitope vaccine (Figure 2). This tool uses a de novo method for structure modeling. GalaxyRefine server was used to refine the final vaccine 3D structure model. The initial generated 3D protein model was refined using GalaxyRefine server. The server-produced five models based on the root- mean-square deviation (RMSD) and MolProbity algorithm (Table 3). Refined model 3, showing the highest Ramachandran value, was selected for performing the docking analysis (Table 3).

**Figure 3:**
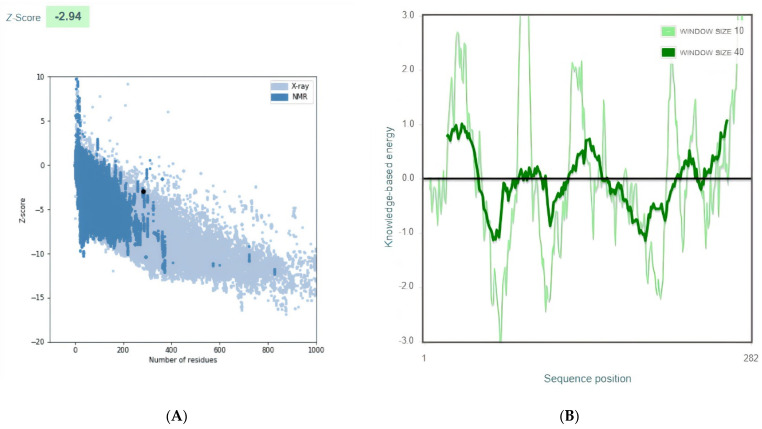
Vaccine 3D Structure Validation by ProSA-web. (**A**): The Z-score of the refined model is −2.94. (**B**): ProSA-web plot of residue scores analysis of the predicted model to check the local model quality and the negative values suggest no erroneous parts of the model structure.

The final multi-epitope vaccine model structures were validated by using ProSA-web. The overall Z-score was −2.94 for the refined model). Negative z-score values indicate no error parts inside the model structure. This score indicates acceptable model quality as it is within the range of the comparable sized native proteins (Figure 3A). The residue scores were checked (Figure 3B)

### 3.6. Molecular Docking Analyses of Multi-Epitope-Based Vaccine against TLR4

**Figure 4:**
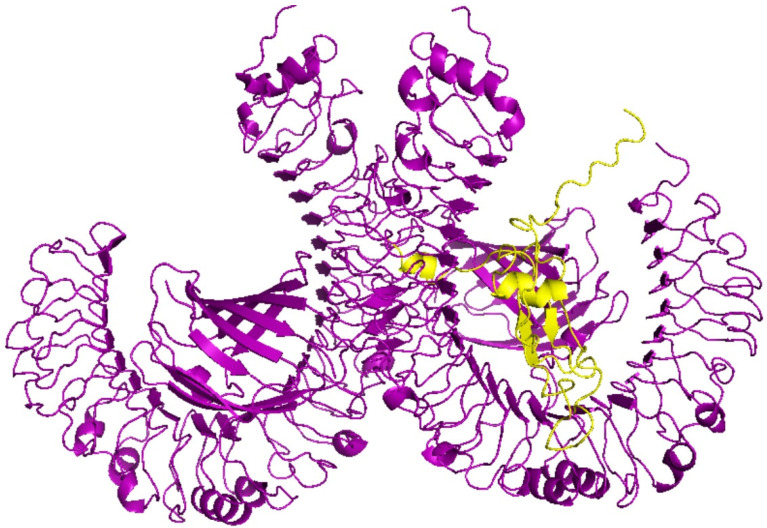
Molecular docking of the vaccine and the TLR4 (PDB ID: 4G8A) immune receptor. Complex of TLR4 (PDB ID: 4G8A) immune receptor in violet and the vaccine in yellow is shown. The score of this complex model is −1346.3, indicating high binding affinity.

Molecular docking of the vaccinse with TLR4 was examined by ClusPro 2.0 server. The antibody mode of ClusPro 2.0 server was selected as this mode was developed for docking antibody and antigen pairs. To improve the docking results, residues that do not fall in the complementarity-determining regions were masked. TLRs have an essential role in innate immunity activation and organization of the adaptive immune response as they detect structurally conserved molecules derived from microbes and viruses [34]. Activation of Toll-like receptor 4 leads to an intracellular signaling pathway NF-κB and inflammatory cytokine production that trigger the innate immune system. The role of TLR4 in the generation of an effective immune response against SARS-CoV-2 has been studied [35]. The 3D model of the vaccine was docked with TLR4 (PDB ID: 4G8A) immune receptor by ClusPro 2.0 server. The lowest energy score, −1346.3, was selected as the best-docked complex, suggesting that the vaccine model occupies the receptor accurately and showing high binding affinity (Figure 4).

### 3.7. Molecular Dynamics Simulation of the Vaccine-Receptor Complex

**Figure 5:**
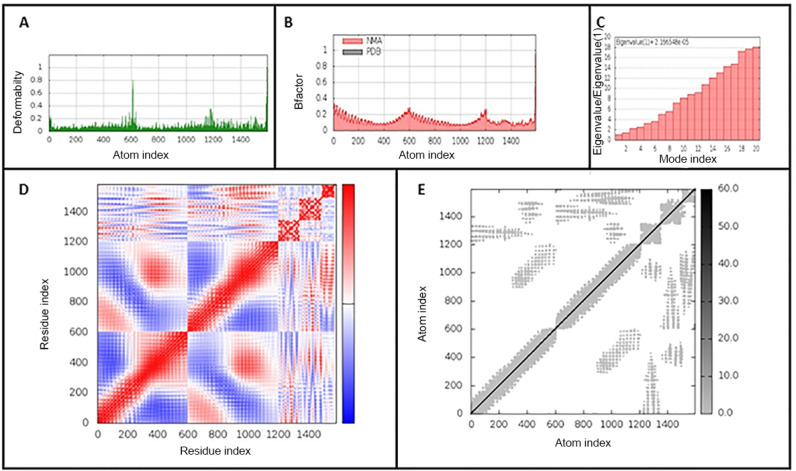
iMODS analysis of the vaccine-TLR4-docked complex. (**A**) Main-chain deformability, (**B**) B-factor values, (**C**) The eigenvalue of the docked complex, (**D**) The covariance matrix between pairs of residues (red: correlated, white: uncorrelated, blue: anti-correlated). (**E**) The elastic network model, suggesting the connection between atoms and springs.

Molecular dynamics simulation was used to study the binding affinity of the vaccine-TLR4 docked complex and to assess its stability and physical movements over time. Analysis of the main-chain deformability and the mobility stiffness of the residues in the complex showed low distortion in the residues of the complex (Figure 5A). The B-factor values were proportional to root mean square (see Figure 5B). B-factor values determines uncertainty of the atomic position, which includes the effects of noise due to model errors and lattice defects, in addition to the positional variance of thermal protein motion. The eigenvalue of the complex is 5.426 × 10^−6^ and it increased gradually in each mode during the dynamics (Figure 5C). The covariance matrix between the pairs of residues is shown in Figure 5D,E shows the elastic network model in the docked complex. The results suggest the stability of the interface between the TLR4-vaccine complex

### 3.8. Codon Adaptation, In Silico Cloning and Testing the Stability of the Secondary Structure of the Produced mRNA

**Figure 6:**
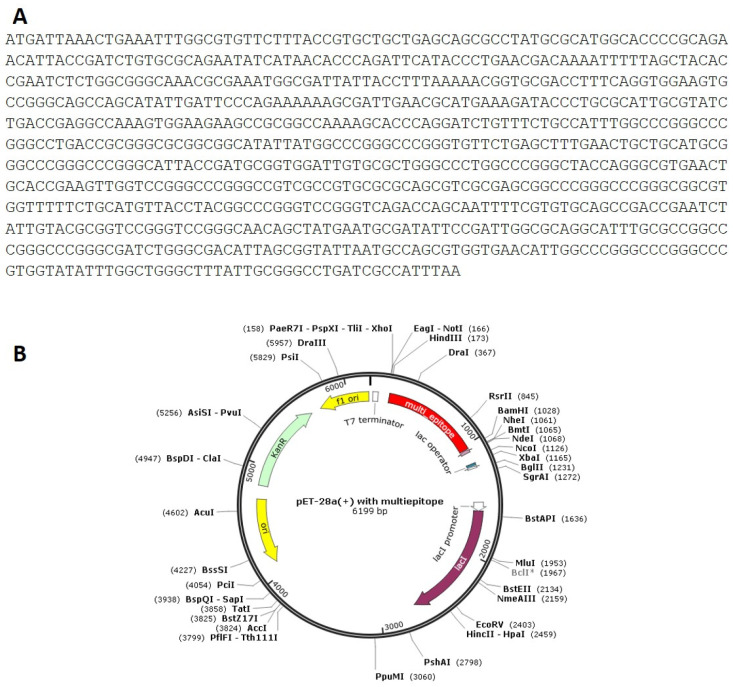
(**A**): Codon adaptation of multi-epitpe to *E. coli* K12 strain. (**B**): Cloning of the designed vaccine the pET-28a (+) vector in silico.

**Figure 7:**
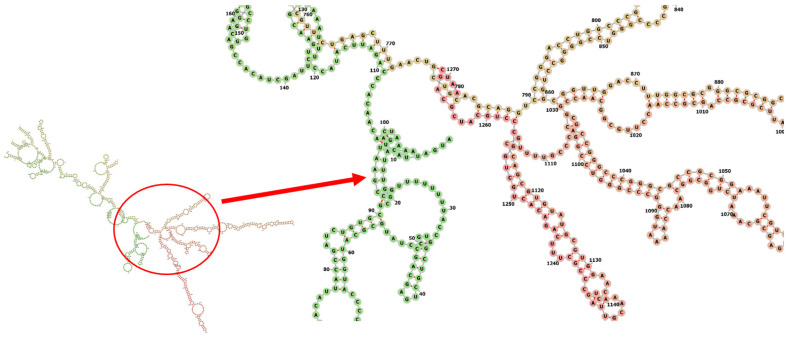
Predicted secondary structure of mRNA for the vaccine.

Codon optimization of the designed multi-epitope was performed to improve the translation efficiency of the construct design. The codons of the designed sequence were adjusted to the *E. coli* K12 on the vector builder service (Figure 6A). The modified nucleotide sequence showed a codon adaptation index (CAI) value of 0.93 guanine-cytosine (GC) content of 59.48%. *Hind* III and *Bam* HI restriction sites were added at the start and end to clone the insert into the pET28a (+) vector. The modified sequence was in silico cloned in the pET28a (+) cloning vector using SnapGene software (Figure 6B). The stability of the secondary structure of the produced mRNA was studied by RNA fold server. The thermodynamic stability of the mRNA structure is indicated by the minimal free energy of −388.60 kcal/mol. The initial 12 nucleotides of the mRNA secondary structure were free of any pseudoknots or long stable hairpins (Figure 7).

## 4. Discussion

Since the release of the first whole genome sequencing of corona virus on February 2020 [36], mutations in the S1 sub-unit of SARS-CoV-2, such as D614G and P681R have developed and become dominant within few months. Investigations suggest a correlation between the D614G mutation and increased viral load, which give the possibility that the D614G mutation increase the infectivity of the virus [7]. Another reports patients carrying strains with G614 have higher airway viral loads but not with severe disease symptoms [37]. On the other hand, another study found a correlation between G614 mutation and higher case fatality rate of COVID-19 [38]. The P681R is a critical mutation as it positioned at the furin cleavage site that splits the S protein to S1 and S2 subunits. It also increases the viral replication of the virus [10].

This study aimed to design a multi-epitope vaccine to the corona virus and more specifically to the corona B.1.617 lineage. The designed vaccine, based on short immunogenic sequences that can generate both humoral and cell mediated immunity. This method of vaccine construction offers improved safety levels and provides the opportunity to specifically attach/engineer combinations of epitopes for augmented potency. This approach offers an alternative for recombinant vaccine technology and spares the use of whole genome/large proteins. This will relieve the extra antigenic load and the allergenic responses [39,40]. Moreover, the conventional method of vaccine designing using large proteins can produce unnecessary antigenic load and may lead to increased chances of allergenic responses [41]. Moreover, this strategy gives the possibility of selecting epitopes producing the anticipated response in largest possible human population [14].

The designed multi-epitope followed the same strategy used by Kar et al. [17]. The multi-epitope was linked at its N-terminal by Cholera Toxin B (CTB) with appropriate linkers. This sequence was verified as a strong viral adjuvant in many studies [42,43,44]. Furthermore, GPGPG was used to join the selected epitopes and make them more reachable. The selected epitopes were confirmed to bind with multiple MHC class I and MHC class II alleles, following the same approach used by Bazhan et al. [45]. One epitope (YQGVNCTEV), showing antigenicity of 1.39 and immunogenicity of 0.08, contains the D614G mutation. The D614G mutation resulted in higher pseudovirus titers in multiple cell types, suggesting that this mutation might be associated with infectivity of the COVID-19 virus and enhanced replication in airways [7,46]. Another epitope (RRRARSVAS), showed antigenicity of 1.16 and immunogenicity of 0.008, contains the P681R mutation. The P681R mutation is the most representative mutation in B.1.617 lineage. Recent studies showed that the D614G/P681R pseudovirus was more resistant to the monoclonal antibodies targeting the RBD domain of the corona virus [9].

The instability index of the designed vaccine yielded 32.35, which predicts that the expressed protein to be stable [27]. The protein is predicted to be highly thermostable as its aliphatic index showed 80.32 [47]. The GRAVY index of vaccine construct was found to be −0.063 (lower the GRAVY score, better is the solubility). These values are similar to the values of Kar et. al. Foroutan et al., used the same sequence of in silico analysis against *Toxoplasma gondii*. They validated the activation of strong humoral and cellular responses by their multi-epitope vaccine in mice by laboratory experiment [48]. They computed the instability index (II) of the vaccine to be 66.37, aliphatic index and GRAVY of multi-epitope peptide were 57.76 and –0.449, respectively. The predicted values of the instability index, aliphatic index and GRAVY were found to be comparable when compared to the previous values. The prediction of the vaccine tertiary structure model was performed using the 3Dpro tool and refined using the ProSA web server and the Z-score assessment yielded a score of −2.94. The obtained Z-score makes our model among the determined structures solved by NMR and X-ray crystallographic experiments.

The binding affinity of complexes of the developed vaccine and TLR4 receptor was confirmed by the ClusPro server. Generation of a stable immune response has to be confirmed, in any in-silico-designed vaccine. TLRs are the main target for these kinds of docking experiments as TLRs have an essential role in innate immunity activation and synchronization of the adaptive immune response against microbes, including viruses [34,49,50]. In this study, TLR4 was selected because of its confirmed role in viral structural proteins recognition leading to inflammatory cytokine production [51]. Moreover, various studies confirmed its role in the generation of an effective immune response against SARs-COV, which makes most in silico approaches test their construct again TLRs [17,50,52,53,54,55,56]. The docking and MD simulation was performed according to Yang et al. [48]. iMOD server was selected for performing the molecular dynamics simulation as it simplifies and creates potential transition pathways between two homologous structures. Toll-like receptor 4 was selected as a target to the designed vaccine as many studies confirm the recognition of the S protein to this receptor [50,57].

The protein sequence of the multi-epitope was reverse translated into its specific cDNA sequence, and its codon was optimized for expression in *E. coli*. Optimization results showed a codon adaptation index (CAI) value of 0.93 and (GC) content of 59.48%. Basically, a (CAI) value > 0.8 and GC content between 30 and 70% are considered efficient for protein expression in the host system [58]. It was in silico cloned into the pET-28a (+) expression vector. We followed the same approach used by Foroutan et al. for the efficient in vitro expression of the vaccine. The allergenecity, physicochemical properties, instability index and aliphatic index of our design are comparable to those predicted by Foroutan et al. [48]. This group has validated their in silico design by laboratory experiments and has confirmed trigging strong humoral and cellular responses in mice.

## 5. Conclusions

Application of immunoinformatic tools in the design of vaccines propose effective vaccines in shorter time with reduced cost. In this report, a multi-epitope SARS-CoV-2 vaccine was designed. Two epitopes carrying the D614G and P681R mutations responsible for increasing the viral infectivity and severity of the disease, were included. The spike multi-epitope protein was in silico tested for the induction of CTLs and HTLs. The designed multi-epitope can be a promising vaccine against strains carrying the any of the two mutations. Despite the high efficacy of the in silico designed candidate vaccine, experimental investigations are needed for its validation.

## Figures and Tables

**Table 1:** (**A**): Cytotoxic T lymphocyte (CTL) epitopes prediction from Spike of SARS-CoV-2. (**B**): Evaluation of SARS-CoV-2 spike protein helper T lymphocyte (HTL) epitopes.

A				
Supertype	Epitope	Position	Antigenicity	Immunogenicity
A1-A2-A3-A24-A26-B7-B7-B27-B39-B44-B58-B62	STQDLFLPF	50	0.6619	0.06828
A1-A2-A3-A24-A26-B7-B7-B27-B39-B44-B58-B62	LTAGAAAYY	258	0.9269	0.15259
A1-A2-A3-A24-A26-B7-B7-B27-B39-B44-B58-B62	VLSFELLHA	510	1.0776	0.1607
A1-A2-A3-A24-A26-B7-B7-B27-B39-B44-B58-B62	ITDAVDCAL	283	0.5260	0.08501
A1-A2-A3-A24-A26-B7-B7-B27-B39-B44-B58-B62	YQGVNCTEV	610	1.3957	0.08675
A1-A2-A3-A24-A26-B7-B7-B27-B39-B44-B58-B62	RRRARSVAS	681	1.1645	0.00854
A1-A2-A3-A24-A26-B7-B7-B27-B39-B44-B58-B62	GVVFLHVTY	1057	1.4104	0.20837
**B**				
**Supertype**	**Epitope**	**Position**	**Antigenicity**	**Immunogenicity**
HLA-DRB1*01:01, HLA-DRB1*03:01, HLA-DRB1*04:01, HLA-DRB1*07:01, HLA-DRB1*08:01, HLA-DRB1*13:01, HLA-DRB1*15:01	QTSNFRVQPTESIVR	312	0.4885	0.1368
HLA-DRB1*01:01, HLA-DRB1*03:01, HLA-DRB1*04:01, HLA-DRB1*07:01, HLA-DRB1*08:01, HLA-DRB1*13:01, HLA-DRB1*15:01	PWYIWLGFIAGLIAI	1211	0.6761	0.81006
HLA-DRB1*01:01, HLA-DRB1*03:01, HLA-DRB1*04:01, HLA-DRB1*07:01, HLA-DRB1*08:01, HLA-DRB1*13:01, HLA-DRB1*15:01	DLGDISGINASVVNI	1163	0.8798	0.10504
HLA-DRB1*01:01, HLA-DRB1*03:01, HLA-DRB1*04:01, HLA-DRB1*07:01, HLA-DRB1*08:01, HLA-DRB1*13:01, HLA-DRB1*15:01	NSYECDIPIGAGICA	656	0.5726	0.50087

**Table 2:** Worldwide human population coverage analysis results using the IEDB population coverage analysis tool.

Population/Area	Coverage
Brazil	85.39%
East Africa	82.0%
England	99.82%
Europe	99.3%
India	88.89%
North Africa	88.79%
Russia	95.21%
Saudi Arabia	95.64%
United States	97.93%
World	96.85%

**Table 3:** Quality scores of the models predicted by GalaxyRefine.

Model	GDT-HA	RMSD	MolProbity	Clash Score	Poor Rotamers	Rama Favored
Initial	1.0000	0.000	3.906	132.2	7.1	83.9
MODEL 1	0.8963	0.550	2.031	15.2	0.5	95
MODEL 2	0.8901	0.540	2.031	15.2	0.5	95
MODEL 3	0.8972	0.554	2.078	14.7	0.9	93.9
MODEL 4	0.8892	0.553	2.040	14.7	0.9	94.6
MODEL 5	0.8963	0.534	2.193	15.4	1.4	94.3

## Data Availability

The datasets used and/or analyzed in the current study are available from the corresponding author on reasonable request.

## Abbreviations

ACE2: angiotensin-converting enzyme 2; CP: cytoplasmic domain; CTB: Cholera Toxin B; CTLs: cytotoxic T lymphocytes; FP: fusion peptide; HR: heptad repeat; HTLs: helper T lymphocytes; Nabs: neutralizing antibodies; NTD: N-terminal domain; pI: isoelectric point; RBD: receptor-binding domain; S protein: spike protein; TM: transmembrane domain; VOC: variants of concern; VOI: variants of interest.
